# Metabolic versatility of freshwater sedimentary archaea feeding on different organic carbon sources

**DOI:** 10.1371/journal.pone.0231238

**Published:** 2020-04-08

**Authors:** Sergi Compte-Port, Mireia Fillol, Frederic Gich, Carles M. Borrego

**Affiliations:** 1 Water Quality and Microbial Diversity, Catalan Institute for Water Research (ICRA), Scientific and Technological Park of the University of Girona, Girona, Spain; 2 Group of Molecular Microbial Ecology, Institute of Aquatic Ecology, University of Girona, Girona, Spain; Universidade Federal do Estado do Rio de Janeiro, BRAZIL

## Abstract

Members of the phylum Bathyarchaeota and the class Thermoplasmata are widespread in marine and freshwater sediments where they have been recognized as key players in the carbon cycle. Here, we tested the responsiveness of archaeal communities on settled plant debris and sediment from a karstic lake to different organic carbon amendments (amino acids, plant-derived carbohydrates, and aromatics) using a lab-scale microcosm. Changes in the composition and abundance of sediment and biofilm archaeal communities in both DNA and RNA fractions were assessed by 16S rRNA gene amplicon sequencing and qPCR, respectively, after 7 and 30 days of incubation. Archaeal communities showed compositional changes in terms of alpha and beta diversity in relation to the type of carbon source (amino acids *vs*. plant-derived compounds), the nucleic acid fraction (DNA *vs*. RNA), and the incubation time (7 *vs*. 30 days). Distinct groups within the Bathyarchaeota (Bathy-15 and Bathy-6) and the Thermoplasmata (MBG-D) differently reacted to carbon supplements as deduced from the analysis of RNA libraries. Whereas Bathyarchaeota in biofilms showed a long-term positive response to humic acids, their counterparts in the sediment were mainly stimulated by the addition of tryptophan, suggesting the presence of different subpopulations in both habitats. Overall, our work presents an *in vitro* assessment of the versatility of archaea inhabiting freshwater sediments towards organic carbon and introduces settled leaf litter as a new habitat for the Bathyarchaeota and the Thermoplasmata.

## Introduction

Archaea are abundant in lake sediments [1–4]. Particularly, members of the phylum Bathyarchaeota and the class *Thermoplasmata* are widespread and considered as core generalists in sediment habitats [5], where they have been recognized as key players in the carbon cycle [6–9]. Archaea are also common components of aquatic biofilms [10–12] and members of the Bathyarchaeota and the Thermoplasmata have been identified in biofilms growing in brackish water springs, acid mine drainages or microbialites [11–14]. Several authors highlighted that archaea thriving in biofilms might also contribute to carbon cycling [12,13] although few data are available regarding their substrate preferences and growth requirements [12–15]. This lack of information is also common for most sedimentary archaea affiliated to lineages without cultured representatives (*i*.*e*. Bathyarchaeota, Thermoplasmata, Thorarchaeota (formerly known as Marine Benthic Group-B, among others) [16,17]. In recent years, the application of metagenomics, single-cell genomics, and stable isotope probing (SIP) provided valuable inferences about the metabolic capabilities of members of archaeal lineages such as the Bathyarchaeota and the Thermoplasmata. For instance, the Bathyarchaeota shows a wide metabolic versatility, ranging from heterotrophy using organic carbon from buried proteins [18] and plant-derived carbohydrates [9] to autotrophy, fixing CO_2_ through either acetogenesis (*via* the Wood–Ljungdahl pathway) [9,19–21] or methanogenesis [22]. More recently, an analysis of 51 metagenomic assembled genomes provided additional support for the metabolic versatility of most subgroups within the Bathyarchaeota [23]. Likewise, several studies demonstrated that members of Thermoplasmata are able to degrade proteins [18], fatty acids [8], and to carry out sulfate reduction [24].

The enrichment and cultivation of members of these uncultured archaeal groups is complex and time-consuming due to the impossibility to mimic all conditions naturally occurring in their habitats [25]. Despite these drawbacks, several research teams have devoted great efforts to enrich and cultivate sedimentary archaea under laboratory conditions. For instance, Gagen and co-workers used a novel cultivation approach to enrich Bathyarchaeota and resolve their tolerance to different oxygen concentrations [15]. Similarly, Seyler and co-workers used ^13^C carbon amendments and SIP to discern the preferences of Bathyarchaeota from salt marsh sediments to different carbon sources [26]. Recently, long-term enrichments set up with marine sediment and amended with different organic compounds provided evidence that Bathyarchaeota subgroup Bathy-8 fixed bicarbonate using energy from the degradation of lignin [27]. This result is in agreement with the identification of genes encoding enzymes involved in the degradation of aromatic compounds in genome fragments from sedimentary Bathyarchaeota [28]. Overall, both cultivation-based studies and genome-centric approaches confirm that some members of the Bathyarchaeota and the Thermoplasmata are able to feed on a wide range of organic compounds.

In the present work, epiphytic biofilms on settled plant debris and sediment were collected from the bottom of a freshwater karstic lake where Bathyarchaeota and Thermoplasmata are prevalent [4,29]. Both type of samples were used to set up laboratory microcosms that were amended with different organic compounds (D- and L-arginine, tryptophan, protocatechuate, humic acids, and pectin) to assess the responsiveness of archaeal groups to these organic carbon sources. We applied a combination of 16S rRNA gene amplicon sequencing and quantitative PCR targeting total Archaea, Bathyarchaeota, and Thermoplasmata in both DNA and RNA fractions extracted after 7 and 30 days of incubation. In biofilms, all target groups responded positively to the addition of amino acids at short-term (7 days of incubation) whereas the stimulation by complex organics (humics and pectin) was only noticeable at long-term (30 days of incubation). In turn, archaea in the sediment were less reactive to organic amendments and only tryptophan caused a positive response in the abundance of Bathyarchaeota and Thermoplasmata. This study presents an *in vitro* assessment of the versatility of archaea inhabiting freshwater sediments towards organic carbon and introduces settled leaf litter as a new habitat for widespread archaeal lineages such as the Bathyarchaeota and the Thermoplasmata.

## Material and methods

### Sample collection and processing

The City Council of Banyoles (http://www.banyoles.cat) and the local environmental agency Consorci de l'Estany (http://consorcidelestany.org/) allowed us the sampling of Lake Cisó by issuing and officially permission for the entire year 2015. The current field study did not involve any endangered or protected species and it was only intended to collect water and sediment samples from the lake. Lake Cisó (42.127634, 2.751717; Banyoles Karstic System) was sampled on 29^th^ January 2015. A depth profile of the main physicochemical variables (i.e. temperature, conductivity, pH, redox potential (*E*_H_) and oxygen concentration) along the water column was recorded *in situ* with a multiparametric probe OTT-HydrolabMS5 (Hatch Hydromet, Loveland, CO, USA) (S1 Fig). Water samples for the determination of sulfide were collected at different depths using a weighted double cone connected to a battery-driven pump as previously described [30]. At each dept, water was collected in sterile screw-capped glass tubes and fixed *in situ* by adding zinc acetate (0.1 M final concentration) under alkaline conditions (NaOH, 0.1 M final concentration). Sulfide concentrations were determined from fixed subsamples according to Brock and co-workers [31]. A 2-liter water sample was also collected from the lake bottom to be used during the set-up of the experimental microcosms (see below).

Surficial sediment and leaf litter settled at the bottom of the lake were collected from the deepest point of the lake (approx. 7.5 m) using an Ekman grab sampler that was deployed three times. On board, collected material was transferred to a sterile plastic tray from where sediment and settled leaves were carefully separated and transferred into sterile glass bottles that were completely filled and immediately capped to minimize exposure to oxygen. Sampling bottles containing sediment and leaf-litter were stored at 4°C into a portable icebox to minimize microbial activity until arrival at the laboratory (within 2 h after collection). Once in the laboratory, a 500 mL sample of the water collected from the lake bottom was filtered through glass fiber filters and then filtered twice through 0.22-μm pore-size, 47-mm-diameter polycarbonate filters (ISOPORE, Millipore, MA). The filtered water (500 mL) was autoclaved, cooled under a N_2_ atmosphere and then amended with 2.5 ml of a sterile 100 mM Na_2_S solution (final concentration 0.5 mM) to mimic *in situ* sulfide concentrations.

### Experimental set-up and inoculation

Since our work was aimed to compare the responsiveness of both sediment and biofilm archaea to different organic carbon compounds, both across treatments (*i*.*e*. carbon sources) and habitats (*i*.*e*. sediment *vs*. biofilm), the preparation of collected material was intended to obtain homogeneous samples that were representative for both habitats to be used as inocula, thus avoiding artifactual biases derived from an heterogeneous composition. All manipulations of collected sediment and leaf litter samples were done using sterile instruments and in an anaerobic chamber (gas mixture N_2_:H_2_:CO_2_ [90:5:5], Coy Lab Products, Michigan, USA) to avoid oxygen exposure during handling. Collected leaves were rinsed with the sterile, pre-filtered, H_2_S-corrected water (rinse water, RW) to remove sediment debris. Biofilm biomass was then carefully detached from the surface of individual leaves (*n* = 25) using sterile cell scrapers (ThermoScientific, Waltham, MA, USA) and collected in sterile 15 mL Falcon tubes. This homogeneous biofilm biomass was then used to inoculate the experimental microcosms (see below). For the preparation of sediment slurries, we first removed the excess of water carried over with the grab sample (≈50 mL) using a sterile Pasteur pipette. A subsample of 10 mL of this overlying water was fixed for sulfide analysis as described above. After removing any plant debris, we prepared a sediment slurry by homogenizing ≈250 mL of bare sediment into a sterile glass beaker. This sediment slurry was then evenly distributed into sterile 15 mL Falcon tubes and then used to inoculate the experimental microcosms (see below).

Incubations were carried out in 24-well sterile plates with lids (Iwaki, Tokyo, Japan, 3.4 mL/well). Two plates (44 wells) were filled with 2.75 mL of sediment slurry and two more plates (44 wells) with 2 mL of the biofilm biomass suspension (S2 Fig). Biofilm-containing wells were then filled with 750 μL of RW to obtain a final volume of 2.75 mL. Negative controls (wells containing sediment or biofilm without any carbon amendment) and blank controls (wells containing 2.75 mL of RW) were distributed in the remaining wells (S2 Fig). The average dry weight of sediment and biofilm inocula was calculated by lyophilizing and weighting eight subsamples prepared with the same volumes used for the inoculation of the microcosms.

Assuming that the target archaeal groups were mainly composed of heterotrophic representatives our main goal was to assess which type of C source could stimulate their activity. The experimental treatments consisted of the addition of different types of organic substrates. Particularly, and bearing in mind that most Bathyarchaeota and Thermoplasmata are heterotrophic anaerobes able to feed on proteins, aromatic compounds, and plant-derived polysaccharides [9,18,28], we used three type of organic substrates, namely: amino acids (L-Tryptophan (108374, Merck), D-Arginine (A2646, Sigma) and L-Arginine (A5006, Sigma-Aldrich), complex organics such as pectin (P9135, Sigma), humic acids (53680, Aldrich), and protocatechuate (37580, Sigma) as aromatic intermediate in the lignin degradation pathway. Stock solutions of each compound were prepared in MilliQ water at the desired concentrations and sterilized by filtration and stored at –20°C until use (S1 Table). Each treatment consisted of six replicate wells that were amended with 250 μL of the corresponding stock solution at day 0 (S1 Table for final concentrations). Inoculated plates were wrapped in aluminium foil to ensure dark conditions and incubated in the anaerobic chamber. After 7 days of incubation, 4 out of the 6 treatment replicates for both sediment and biofilm (including non-amended and water internal controls) were collected using sterile pipettes, distributed in properly labelled sterile Eppendorf tubes, and immediately stored at –80°C until nucleic acid extraction. Same procedure was carried out for the remaining two replica treatments after 30 days of incubation.

### Nucleic acid extraction

Samples of sediment and biofilm from all treatments and time points were thawed at room temperature and used for both RNA and DNA extraction using PowerSoil^®^ Total RNA Isolation Kit and RNA Powersoil^®^ DNA Elution Accessory Kit, respectively (MoBio Laboratories, Solana Beach, CA, USA) following manufacturer’s instructions and including an homogenization step (3x) by bead-beating (FastPrep^®^-24 Classic, MP Biomedicals, CA, USA). Water samples from blank controls were also thawed at room temperature and filtered through 0.22 μm pore size 47-mm diameter polycarbonate filters (ISOPORE, Millipore, MA, USA) that were further used for DNA and RNA extraction as described for sediment and biofilm samples. Concentration of DNA and RNA in resulting extracts was determined using QUBIT^®^ 2.0 Fluorometer (Invitrogen Molecular proves Inc., Oslo, Norway). TURBO DNAfree^™^ Kit (Ambion, Austin, TX, USA) was used to remove DNA traces in RNA extracts. Reverse transcription of RNA to cDNA was done using random hexamer primers and Superscript III First-Strand Synthesis System (Invitrogen, Carlsbad, CA, USA) according to manufacturer’s instructions. Concentration of cDNA was also quantified using Qubit^®^ 2.0 Fluorometer.

### 16S rRNA gene amplicon sequencing and processing

High-throughput multiplexed 16S rRNA gene sequencing with the Illumina MiSeq System (2×250 PE) was carried out using the general archaeal primer pair 519f/1017r [32,33] complemented with Illumina adapters and sample-specific barcodes at the genomics core facilities of the Research Technology Support Facility Michigan State University, USA [34]. On average, each sample was sequenced at a depth of 143 K reads, accounting for 12 million reads for the overall analysis. By using the 2x250 PE Illumina chemistry, the resulting forward and reverse sequences produced by this primer combination did not completely overlap thus causing a fail of the pair merging procedure. We thus decided to analyze separately 5’ (forward) and 3’ (reverse) reads using the same bioinformatic pipeline and comparing the results using Procrustes [35]. Both sequence datasets were quality filtered, chimera checked and clustered into Operational Taxonomic Units (OTU, 97% cutoff) using default parameters in QIIME v. 1.9.1 [35]. QIIME was also used to construct the correspondent OTU tables, to align the representative OTU sequences against the Greengenes imputed core reference alignment [36], and to assign the taxonomic information to each OTU using the BLAST method and the QIIME-formatted version of the SILVA 123 reference database. Four samples (SDA3, ST1, SLA1 and SC1 from the DNA library) yielded a sequence coverage < 10,000 reads and they were discarded from downstream analyses. QIIME was also used to compute alpha diversity indicators of richness and diversity after rarefying OTU tables to 12,000 sequences per sample to standardize sequencing effort across samples. The community similarity among sites (beta diversity) was calculated using unweighted and weighted UniFrac distances [37]. To test whether the conclusions from the analysis of beta diversity were essentially identical regardless of the sequence dataset used (5’ or 3’), we ran Procrustes analysis in QIIME using the unweighted and weighted UniFrac distance matrices and 10,000 MonteCarlo simulations. Procrustes results confirmed that the same conclusions could be derived with either dataset (M^2^ = 0.040, *p* < 0.001 for unweighted UniFrac; M^2^ = 0.186, *p* < 0.001 for weighted UniFrac, S3 Fig). Since the average quality of the forward reads (5’) was slightly higher than those obtained from reverse reads (3’) (average Q-score of R1 and R2 reads was 31.9 and 28.6, respectively), all downstream analyses were conducted on the forward sequence dataset. Due to lack of database information, the phylogeny for the phylum *Bathyarchaeota* was resolved by adding the Bathyarchaeota reads to a previously built Neighbor-Joining phylogenetic tree (ARB software; Ludwig 2004) composed of reference sequences of the 21 Bathyarchaeota subgroups [5]. The raw sequencing data of this study have been deposited in the NCBI short-read archive (SRA) under accession numbers SAMN08954527 to SAMN08954697.

### Quantification of 16S rRNA genes by qPCR

Copy numbers of 16S rRNA molecules and genes from Archaea, Thermoplasmata and Bathyarchaeota were determined by qPCR using specific primers (R^2^, efficiencies and assay conditions are summarized in S2 Table). Quantifications were performed in a Mx3005P system (Agilent Technologies) using SYBR Green detection chemistry, qPCR 96-well plates (Agilent Technologies, Cat. N° 401334), and Mx3000P Optical Strip Caps (Agilent Technologies, Santa Clara, CA, USA Cat. N° 401425). Every reaction was prepared for a final volume of 30 μL containing 15 μL of 2× Brilliant III Ultra-Fast SYBR Green qPCR Master Mix (Agilent Technologies), 1.2 μL of each forward and reverse primer (final concentration of 4 μM), 1 μL of template DNA and molecular biology grade water up to 30 μL volume. Standards for archaeal 16S rRNA gene quantification were made using genomic DNA from *Sulfolobus solfataricus* DSM1616. Plasmids containing inserts of environmental 16S rRNA gene fragments previously isolated from clone libraries [29] were used as standards for the quantification of Bathyarchaeota and Thermoplasmata. Standard curves were obtained after serial dilutions of the corresponding DNA extracts containing known concentration of target gene ranging from 10^9^ to 10^2^ gene copies per μl. Data analyses were carried out using MxPro qPCR Software for Mx3005P qPCR System (Agilent Technologies). All qPCR analyses carried out followed the MIQE rules for qPCR analyses [38].

### Statistical analyses

The rest of the work was conducted using R software [39]. Due to the lack of normality found in Shapiro-Wilk tests, significant differences (*p* < 0.05) for alpha diversity estimators were assessed by Mann-Whitney non-parametric test (Package “stats” version 3.4.0). The OTU contingency table together with the SINA taxonomy file, the phylogenetic tree, following the algorithm described by Price and co-workers [40], and the metadata file (classifying the samples among the different categories: DNA/RNA, Sediment/Biofilm, carbon compound, amendment type, and time) were imported into R to run beta diversity and statistical analyses using packages *phyloseq* ([41]; version 1.14.0) and *vegan* ([42]; version 2.3–5). Raw data was rarefied (12,000 sequences/sample). A distance matrix (Bray-Curtis dissimilarities on OTU abundances) was calculated and samples were ordinated using non-metric multidimensional scaling (NMDS) either by considering the sample set as a whole or subsets defined by treatments or groups of interest. Homogeneities in beta dispersion (*i*.*e*. ANOVA for distances from data points centroid; *p*-value = 0.05) were assessed through experimental groups by the function betadisper(). Changes in community composition were assessed by function adonis(), which determined: *i)* the significant effects (*p*-value < 0.05) of factor levels over composition, and *ii)* the amount (%) of variance on the OTU contingency table explained by these factors. To estimate which lineages significantly contributed to compositional changes across experimental factors (*p-*value < 0.05), we used the function simper() in package *vegan* (similarity percentage, [43]) to find the average contribution of each lineage to the average overall Bray-Curtis dissimilarity between pairs of samples. In PERMANOVA and SIMPER analyses, *p-*values were obtained after 9,999 permutations.

## Results

### Physicochemistry of the water column

The water column of Lake Cisó was thermally and chemically stratified around 3 m depth on the day of sampling (S1 Fig). Despite the stratification, the whole water column was completely anoxic and only a slight oxygenation was measured at the first 0.5 m. Sulfide was present in the whole water column increasing its concentration in depth and reaching its maximum at the lake bottom (765 μM). In lake Cisó, these euxinic conditions (anoxic and sulfide rich) are maintained during the whole year although summer stratification slightly oxygenates the surficial water layer creating a narrow, warmer and oxygen-deficient epilimnion [4]. Overall, the physical and chemical conditions of the water column at the day of sampling confirmed the euxinic conditions at the lake bottom. Although we are aware that some oxygenation of the collected material (sediment and settled leaves) might have occurred during handling and transport, the overlying water carried over with the sediment was still anoxic and sulfide-rich (average of replicate measurements was 400.9±108.8 μM, S3 Table). We thus assumed that this oxygenation was minimal during collection and insufficient to critically alter the composition of archaeal communities in both type of samples according to the comparison of relative abundances of archaeal groups in the collected material (see below) with those previously described for sediments from the lake [4] and in biofilms from settled leaves (Fillol, unpublished results).

### Composition of the inocula

Archaeal communities in source biofilm and sediment samples showed compositional differences in both DNA and RNA libraries (S4 Fig). In biofilms, the bulk archaeal community was mainly dominated by sequences affiliated to Thermoplasmata (42% of total reads), Woesearchaeota (superphylum DPANN, 29%) and Bathyarchaeota (16%). Similar relative abundances were measured for the same groups in RNA library (35%, 29% and 20%, respectively). In the sediment, the relative abundance of these groups in the bulk community (DNA fraction) was also similar to those in biofilms except for the Bathyarchaeota, which were less abundant (9% of total reads) (S4 Fig). In the sediment RNA library, sequences affiliated to Thermoplasmata decreased in their relative abundance (33% of total reads) compared to DNA (45%) whereas Woesearchaeota exhibited the inverse trend (37% in RNA and 28% in the DNA library). Concerning Bathyarchaeota, no difference was observed between both libraries (9%). Assuming this archaeal composition as starting point, we then assessed if either incubation time (7 *vs*. 30 days) or treatments (different carbon sources) caused variations on both the overall composition (at the phylum level) and the microdiversity (at the OTUs level) of archaeal communities in DNA and RNA fractions of biofilms and sediments.

### Changes in the composition of DNA and RNA archaeal communities according to habitat and incubation time

**Fig 1:**
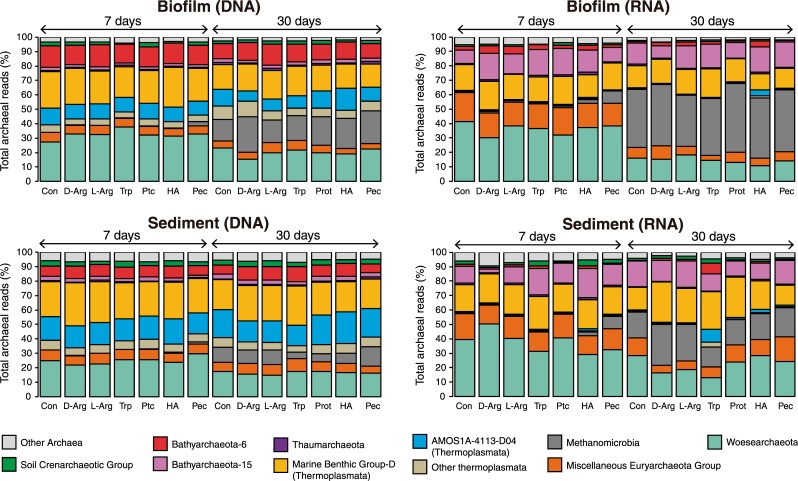
Average relative abundance of different archaeal taxa in DNA (left) and RNA (right) libraries from biofilm (upper panels) and sediment (lower panels) after incubation for 7 days (*n* = 4) and 30 days (*n* = 2) under the different treatment conditions. Con: Control (no addition of organic carbon); D-Arg: D-Arginine; L-Arg: L-Arginine; Trp: Tryptophan; Ptc: Protocatechuate; HA: Humic Acids; Pec: Pectin. The relative abundance of each phylogenetic group is depicted as a percentage of total reads. NOTE: the taxonomical level is not consistent across the displayed taxa to better illustrate changes in the abundance of groups within the phylum Bathyarchaeota and the class Thermoplasmata.

Experimental treatments did not cause remarkable changes in the overall composition of archaeal communities, which mainly retained the relative contribution of the three main lineages (Thermoplasmata, Woesearchaeota, and Bathyarchaeota) identified in the source communities (biofilm and sediment samples used as inoculum). Members of the three lineages remained prevalent during incubation time under all treatments (Fig 1 and S4 Fig). The Thermoplasmata community was mainly composed of sequences affiliated to the Marine Benthic Group-D (MBG-D) and the AMOS1A-4113-D04, while Bathyarchaeota reads affiliated to subgroups Bathy-6 and Bathy-15. Both subgroups exhibited great disparities in their relative abundances between DNA and RNA fractions. Whereas Bathyarchaeota-6 was well represented in DNA extracts from biofilm and sediment samples (between 5% and 18.4% of total archaeal reads; Fig 1), they represented just a minor fraction in the RNA library (2.2 ± 1.5% of reads). Conversely, subgroup Bathy-15 was among the most abundant groups in the RNA fraction (14.5% ± 3.5% and 13.7% ± 4.4% in biofilm and sediment samples, respectively; Fig 1). Other groups that increased their relative abundance in the RNA library compared to the DNA library were the Miscellaneous Euryarchaeota Group (MEG; 6.3 ± 1.2% and 13.5 ± 5.2% of reads in DNA and RNA, respectively) and the AMOS1A-4113-D04 (13.2 ± 3.8% and 1 ± 1.5% of reads in DNA and RNA, respectively both affiliated to Class Thermoplasmata; Fig 1).

To better discriminate the potential changes caused by experimental treatments on the composition and abundance of archaeal groups, OTU tables were further filtered according to library (DNA and RNA), habitat (biofilm and sediment), and incubation time (7 days *vs*. 30 days, which reflected short *vs*. long term effects) and compared separately in terms of alpha and beta diversity.

Bulk archaeal communities (DNA fraction) showed a significantly higher richness and diversity (Observed OTUs and Shannon index, respectively) compared to their RNA fraction (Mann-Whitney test; *p* < 0.001; S5A Fig). Differences were also observed when RNA samples were compared according to their habitat type (biofilm *vs*. sediment), being biofilm communities richer and more diverse than those in the sediment (Mann-Whitney test; *p* < 0.044 and *p* < 0.018 for OTU number and Shannon index, respectively; S5B Fig).

Beta diversity analysis further highlighted the differences between the DNA and the RNA fractions of archaeal communities. Indeed, separation by nucleic acid (DNA *vs*. RNA) explained a 44% of compositional variance (PERMANOVA; *p <* 0.0001; S6A Fig). Particularly, Woesearchaeota, Thermoplasmata (MBG-D and AMOS1A-4113-D04), Bathy-6, and Bathy-15 significantly contributed to these changes (PERMANOVA; *p* < 0.001 for all cases, S6A Fig). Again, significant differences in the composition of RNA archaeal communities were observed between biofilm and sediment habitats. In the latter, the main contributors to the explained variance were methanogenic lineages (Methanobacteriales and Methanococcales, S6B Fig). Incubation time was also a prominent factor affecting the alpha and beta diversities of RNA archaeal communities. Richness decreased after 30 days of incubation when compared to 7-days (Mann-Whitney test; *p* < 0.001 in biofilm and sediment; S5C and S5D Fig). Time also had a severe effect on the community composition from both habitats (PERMANOVA; *p* < 0.001 in both cases), explaining a 74% and a 43% of the variance in biofilm and sediment communities, respectively (PERMANOVA; *p <* 0.0001 S5C and S5D Fig). A conspicuous increase in the relative abundance of sequences affiliated to methanogens (*e*.*g*. *Methanomicrobia*) was detected in the RNA fraction of biofilm communities through time (from 2.4 ± 3.3% after 7 days to 41.9 ± 5.4% after 30 days). The same trend but less evident was observed in the archaeal communities from sediments (Fig 1).

### Effect of experimental treatments on the microdiversity of archaeal communities

Due to the lack of clear effects of carbon amendments on the overall community composition (S5 Table), PERMANOVA was further used to check for variations in the abundance of OTUs (*i*.*e*. microdiversity) affiliated to target lineages in RNA libraries from biofilm and sediment in relation to the type of carbon supplement (amino acids *vs*. plant-derived compounds). Significant *p*-values from PERMANOVA tests ruled out that these variations were caused by chance (S7 and S8 Figs). The homogeneity of dispersion (*i*.*e*. beta-dispersion) among the tested groups was also verified to discard confounding effects in PERMANOVA tests. Three groups (Bathy-6, MBG-D, and the Woesearchaeota), exhibited significant differences in microdiversity while maintaining their homogeneity of dispersion, indicating non-random variations of OTU abundances in relation to the type of C amendment. In parallel, the ordination of samples using non-metric multidimensional scaling (NMDS) was done to better identify the clustering of samples on the basis of OTU profiles according to treatment factors (S7 and S8 Figs).

In biofilms, subgroup Bathy-6 showed variations related to the type of compound after both 7 (PERMANOVA; *p* < 0.001) and 30 (PERMANOVA; *p* = 0.032) days of incubation. The type of the carbon source explained 46% and 37% of the variance of OTUs affiliated to subgroup Bathy-6 after 7 and 30 days, respectively (S7 Fig). Same responsiveness was also found in biofilms for MBG-D (PERMANOVA; *p* = 0.037) and Woesearchaeota (PERMANOVA, *p* < 0.001) but only at long-term (*i*.*e*. 30 days of incubation, S7 Fig). In sediments, MBG-D (PERMANOVA, *p* < 0.044) and Woesearchaeota (PERMANOVA, *p* < 0.016) were also responsive to treatments after 30 days of incubation (S8 Fig).

### Stimulation of target groups by carbon amendments

**Fig 2:**
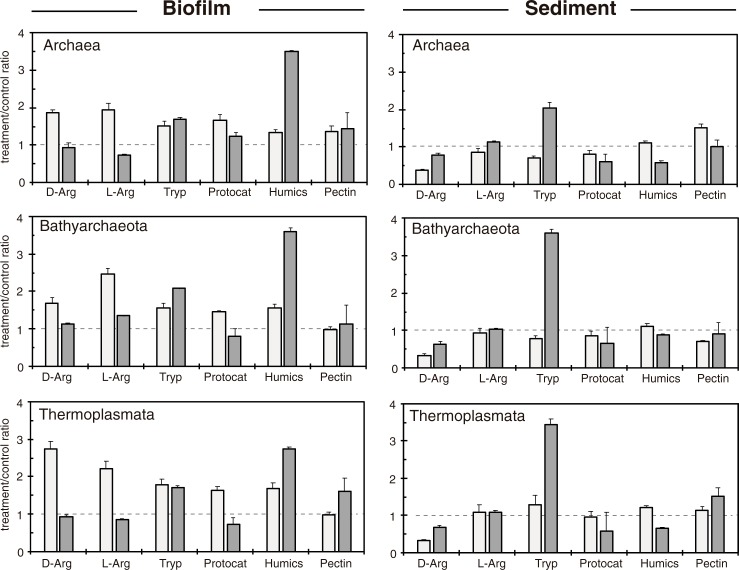
Variation in the ratio between the number of 16S rRNA copies (per ng of RNA) of target groups in each experimental treatment and that in unamended controls. A ratios >1 (dashed line) indicates a positive response to the treatment (*i*.*e*. carbon source). Bars show the mean (±SEM) of short-term (7 days, *n* = 4, light gray) and long-term incubations (30 days, *n* = 2, dark gray).

We calculated the ratio between the copy number of 16S rRNA molecules from target groups (Archaea, Bathyarchaeota and Thermoplasmata) in RNA extracts from treatment wells to those in the control (non-amended wells) as a rough estimate of the potential stimulation of a given taxa across treatments and incubation time (Fig 2). We used this ratio instead of the more popular 16S rRNA/DNA to avoid problems derived from different growth strategies, cell ribosome content, and relic DNA in sediments [44–46]. In biofilms, almost all target groups showed a positive response to all carbon compounds at short-term (7 days of incubation) but only some of them (tryptophan, humic acids, and pectin) sustained this positive stimulation at long-term (30 days) (Fig 2, left panels). Remarkably, archaea were stimulated both by amino acids (including D forms), by aromatic compounds (protocatechuate, humic acids), and polysaccharides (pectin) but only the latter were able to sustain this stimulation after 30 days of incubation (Fig 2). This response was not observed for the archaea in the sediment, where only tryptophan caused a positive response at long-term (Fig 2, right panels). The differential stimulation of Bathyarchaeota and Thermoplasmata between the biofilm and the sediment suggested that both habitats were probably occupied by different populations.

## Discussion

### Methodological considerations

The use of homogeneous biofilm and sediment samples for the inoculation of microcosms impeded any attempt to resolve compositional differences of source archaeal communities at the spatial scale. As stated previously (see Material & Methods), collection and preparation of inocula was intended to obtain homogeneous, representative samples for both habitats to assess how biofilm and sedimentary archaea respond at short and long-term to organic carbon amendments. Thus, it is important to remark that our experimental design lacked true replication since only single composite samples of both sediment and biofilm were compared despite every treatment was assayed using replicates (n = 4 and n = 2 for short- and long-term intervals). Besides, treatment wells were not randomized in the incubation 24-well plates used as microcosms. Although this can be considered a lack of interspersion, the small size of these plates and the homogeneous conditions within the anaerobic chamber used for incubation minimized the risks of confusing results due to random variability among experimental units. Finally, we decided to incubate the microcosms for 30 days without replenishment of the added organics. This decision was made as a trade-off between an optimal time interval to observe changes in the composition of archaeal communities according the large variability in doubling times estimated for most Bathyarchaeota (from days to months, [26,27,47–49]) and the minimal disturbance of the microcosms during incubation. Accordingly, it is important to bear in mind that a potential shortage of added substrates might have occurred during incubation. Despite these considerations, results demonstrated that the observed changes were not caused by random variation but by a differential response of target archaeal groups to the type of organic amendments thus suggesting that the effect of such substrate limitation, if any, was negligible.

### Responsiveness of Bathyarchaeota to organic carbon amendments

Despite that the different treatments assayed did not produce prominent changes in the overall community composition, significant and non-random variations were observed for the Bathyarchaeota, the Thermoplasmata, and the Woesearchaeota at OTU level, thus suggesting a differential response to the tested carbon sources. This observation was further supported by the observed decrease in the overall richness of the archaeal community in the RNA fraction, which suggests that fewer taxa remained active [50]. The comparable reduction in diversity also pointed out to a specialization of the community towards archaeal groups whose members were able to sustain some activity through time under the conditions imposed.

The similar response of Bathyarchaeota and Thermoplasmata to all carbon sources points to similar substrate preferences and agrees with the co-occurrence of both groups in sediments worldwide [4,29]. The ability of Bathyarchaeota to feed on a wide range of organic substrates has already been observed in enrichment cultures [26]. Very recently, Yu and co-workers demonstrated that some members of the Bathyarchaeota were able to grow organoautotrophically using lignin and bicarbonate as energy and carbon source, respectively [27]. Authors measured a 10-fold increase in the copy number of Bathyarchaeota 16S rRNA in long-term enrichments (12 months) set up with marine sediment and supplemented with lignin whereas other organic substrates showed little or no influence on their abundance. This particular response was mainly due by the grow of a population of Bathy-8, a subgroup of Bathyarchaeota that is prevalent in marine sediments [4,27,51]. Although less prominent, we observed a 4-fold increment in the copy number of bathyarchaeotal 16S rRNA in biofilms amended with humic acids (Fig 2). Since humic acids are known to contain polyphenolic derivatives from lignin at different proportions [52,53], the degradation of lignin and their derivatives under anoxic conditions may be plausible not only for subgroup Bathy-8 [27,28] but also for members of other bathyarchaeotal subgroups such as the Bathy-6 and the Bathy-15. Indeed, genes involved in the degradation of benzoate (a derivative of the lignin degradation pathway) have recently been identified in metagenomic assembled genomes from most Bathyarchaeota subgroups, including Bathy-6 and Bathy-15 [23]. Moreover, subgroup Bathy-6 is widespread in freshwater sediments having high C/N ratios (indicator for allochthonous vegetal inputs, [54]). This observation also agrees with the capacity of members of this group to degrade plant-derived polysaccharides of different complexity [9]. The short-term response (7 days of incubation) of Bathy-6 to organic carbon amendments in biofilms also agree with their fast doubling times estimated in ^13^C-labeling experiments (2–2.5 days [26]) although other studies measured doubling times ranging from 1 to 3 months [27,47–49]. We could not formally ruled out, however, the possibility that members of the Bathyarchaeota (including subgroup Bathy-6) assimilated simple C1-compounds derived from bacterial or archaeal metabolism (heterotrophy-based acetogenesis, [55]) or fixed CO_2_ using the reductive acetyl-CoA pathway (i.e. Wood-Ljungdahl) [9]

In turn, members of subgroup Bathy-15 were scarce in our DNA libraries (i.e. bulk fraction of the community) but prevalent in RNA libraries from all experimental treatments (Fig 1). Assuming that a higher abundance of gene signatures for a given group in the RNA extracts compared to non-supplemented controls may reflect differences in activity caused by treatment, the positive stimulation of Bathy-15 to all carbon amendments (complex polysaccharides, amino acids, and aromatics) points towards their metabolic versatility either using a wide range of organic substrates or carrying out acetogenesis [23]. The four-fold increase in bathyarchaeotal 16S rRNA gene copies in sediments amended with tryptophan after 30-days of incubation in comparison to amended biofilms, suggests that both habitats might contain distinct populations differing both in their substrate preferences and in their physiological requirements.

### Responsiveness of Thermoplasmata to organic carbon amendments

Compared to Bathyarchaeota, less information is available for members of the Thermoplasmata. The faster response of biofilm-dwelling Thermoplasmata to amino acids contrasted with the long-term response in treatments supplemented with plant-derived compounds. The higher bioavailability of amino acids compared to more complex, recalcitrant plant-derived compounds may explain this delay [56,57]. Similar to Bathy-6, MBG-D exhibited changes in its microdiversity in relation to the type of carbon source (S7 and S8 Figs). Several studies have reported the genetic potential of members of the MBG-D for the degradation of simple molecules through fermentation or acetogenesis [18,24,58]. Moreover, the stimulation of Thermoplasmata in treatments supplemented with amino acids (Fig 2) agrees well with previous genomic surveys that identified genes encoding for extracellular proteases in genomes from MBG-D [18]. The positive response of biofilm-dwelling Thermoplasmata to D-Arginine could also suggests their ability to incorporate D-enantiomers (as reported for the Bathyarchaeota, [18]). Alternatively, MBG-D may feed either on by-products from archaeal/bacterial metabolism as previously suggested [18] or on bacterial debris, which are rich in D-forms [59,60].

### Responsiveness of other archaeal groups

The Woesearchaeota (formerly known as DHVEG-6) also accounted for an important fraction of archaea in the surficial sediment of Lake Cisó. The Woesearchaeota is a genetically diverse phylum mainly composed of heterotrophic representatives [24,61,62]. In freshwater environments, their members are reactive to phosphate [62] and involved in biomass transformations [61–63]. The analysis of nearly completed genomes of member of this phylum identified a wide variety of proteases and peptidases [61] thus providing an explanation for their long-term responsiveness to amino acid amendments.

Finally, it is worth mentioning the increase in the abundance of methanogenic archaea (*e*.*g*. Methanomicrobia) in all treatments after 30 days of incubation considering their low representativeness in the source lake [4,29]. In fact, Methanomicrobia were outstanding contributors for time-related compositional changes (especially in biofilms; Fig 1 and S5 Fig). The decrease in the amount of 16S rRNA molecules for biofilm-dwelling Woesearchaeota in RNA fractions after 30 days of incubation (Fig 1) might partially explain the relevance of *Methanomicrobia* in terms of relative abundance and its contribution to these diversity changes (S6 Fig). The increase in the relative abundance of methanogens during incubation time may be explained by the production of simple organic compounds as by-products of bacterial or archaeal metabolism accompanied by the depletion of sulfate in the experimental treatments, the latter causing a reduction in the activity of sulfate reducing bacteria while fostering methanogenesis [64–66]. In sediments from karstic lake Cisó, methanogens are outcompeted by sulfate reducers, which are fueled by the constant input of high sulfate concentrations (0.8 g L^–1^) in phreatic water [67]. Considering the fast turnover rates of methanogens [68,69], it would be tempting to speculate that archaeal groups prevalent under natural conditions were outcompeted by methanogens under artificial conditions imposed in the laboratory microcosms.

Overall, the results from our experimental microcosms confirm the metabolic versatility of members of archaeal phyla Bathyarchaeota, Thermoplasmata, and Woesearchaeota towards different organic carbon sources and suggest that these archaeal groups may play a role in the mineralization of labile and recalcitrant organic compounds in euxinic sediments and settled plant litter. Development of innovative cultivation strategies in combination with direct measurements of activity at single cell level (e.g., NanoSIMS) and metagenomic approaches are needed to unequivocally resolve carbon fluxes between these archaeal groups, the conditions under which some pathways are favored, and to corroborate the syntrophic relationships that may occur in these habitats between microbial counterparts.

## Supporting information

S1 Table Organic substrates used for the amendments with the final concentration (in the plate wells) of the different compounds. (DOCX)

S2 Table Primer pairs and conditions used for the quantitative PCR. In all cases denaturalization stages of 95°C for 20 seconds and annealing steps of 60 seconds were performed. Efficiencies and R2, of the standard curves are displayed as intervals
(DOCX)

S3 Table Concentration of sulfide in water from the lake bottom, the overlying water carry over with the collected sediment and the rinse water (RW) used for setting-up the experimental microcosms. Sulfide concentration was determined according to Brock et al (*J*. *Bacteriol*. 1971, 107:303–314) after fixation of sulfide using zinc acetate (see Material and Methods for details). Values are the mean of replicate measurements ± standard deviation.
(DOCX)

S4 Table Mean relative abundance of archaeal groups in libraries from biofilm and sediment after 7 (n = 4) and 30 days (n = 2) of incubation under treatment conditions. CNT: Control (no addition of organic carbon); D-Arg: D-Arginine; L-Arg: L-Arginine; Trp: Tryptophan; Ptc: Protocatechuate; HA: Humic Acids; Pec: Pectin.
(DOCX)

S5 Table Copy numbers of the 16S rRNA gene (normalized by dry weight) for Archaea, Bathyarchaeota and Thermoplasmata. Values are displayed as the average ± standard deviation of biological replicates (*n* = 4 for 7 days and *n* = 2 for 30 days of incubation) which share same levels across experimental factors.
(DOCX)

S1 Fig Depth profile of temperature (red circles), conductivity (black squares), dissolved oxygen (blue circles), oxidation-reduction potential (grey triangles) and sulfide concentration (yellow diamonds) in Lake Cisó at the day of sampling (29/01/2015). (DOCX)

S2 Fig Schematic representation of the sampling procedures, experimental setup for the preparation of microcosms and the list of carbon compounds (treatments). RW: rinse water (see main text for details).
(DOCX)

S3 Fig PCoA Ordination of samples according to (A) unweighted and (B) weighted UniFrac distance using forward (5’) and reverse (3’) sequences. Samples are coloured according to substratum, biofilm (red) and sediment (blue). Samples derived from forward and reverse sequencing are linked with a bar: in every case, the distance between the 5′ and 3′ reads of the same samples is much smaller than the distance between samples. Results from the Procrustes analysis are also shown for each case (10,000 Monte Carlo simulations).
(DOCX)

S4 Fig Composition archaeal communities in (A) biofilm and (B) sediment samples used as inoculum of experimental microcosms. The relative abundance of each taxon is depicted as a percentage of total reads.
(DOCX)

S5 Fig Comparison of alpha diversity estimators (number of OTUs and Shannon index) of biofilm and sediment archaeal communities between (a) DNA and RNA libraries; (b) between niches (biofilm vs. sediment) for archaeal communities in the RNA library; (c) incubation time (0, 7, and 30 days) for biofilm communities in the RNA library; (d) incubation time (0, 7, and 30 days) for sediment communities in the RNA library. Significant differences for all comparisons have been assessed by Mann-Whitney test and indicated by letters above boxplots with correspondent p values.
(DOCX)

S6 Fig Non-metric multidimensional scaling (NMDS) ordination of samples according to community composition (Bray-Curtis dissimilarity distance). Comparison has been done according to: (a) nucleic acids; (b) habitat (only the archaeal community in the RNA library); (c); incubation time (only the biofilm archaeal community in the RNA library); (d) incubation time (only the sediment archaeal community in the RNA library). Significant differences for all comparisons have been assessed by PERMANOVA and beta dispersion analyses (9,999 permutations). *p-*values of both tests are shown at the bottom right of each plot, together with the amount of variance (%) explained by each grouping. Prominent archaeal lineages contributing to such differences have been identified using SIMPER analysis (9,999 permutations) and results are shown in the top right of each plot when appropriate.
(DOCX)

S7 Fig Non-metric multidimensional scaling (NMDS) ordination of samples from microcosms inoculated with biofilm material according to the abundance of OTUs affiliated to Bathyarchaeota (upper panels), MBG-D (middle panels), and Woesearchaeota (lower panels) in RNA libraries after 7 days (left) or 30 days (right) of incubation. Samples are colored according to the carbon source, namely: amino acids (grey squares), plant-derived compounds (black squares) and no carbon addition (white squares). *p*-values from the PERMANOVA and the beta dispersion tests and the amount of explained variance by amendment type (%) are also shown. See main text for details.
(DOCX)

S8 Fig Non-metric multidimensional scaling (NMDS) ordination of samples from microcosms inoculated with sediment material according to the abundance of OTUs affiliated to MBG-D (upper panels) and Woesearchaeota (lower panels) in RNA libraries after 7 days (left) or 30 days (right) of incubation. Samples are colored according to the carbon source, namely: amino acids (grey squares), plant-derived compounds (black squares) and no carbon addition (white squares). *p*-values from the PERMANOVA and the beta dispersion tests and the amount of explained variance by amendment type (%) are also shown. See main text for details.
(DOCX)
