# Supplementary material for: Metabolic versatility of freshwater sedimentary archaea feeding on different organic carbon sources
Source: PLoS One. 2020 Apr 8;15(4):e0231238. doi: 10.1371/journal.pone.0231238 (PMC7141681; doi:10.1371/journal.pone.0231238)
Supplement: S1 Table — (DOCX) [file pone.0231238.s001.docx]

**Supplementary Table S1.** Organic substrates used for the amendments with the final concentration (in the plate wells) of the different compounds.

|  | **Compound** | **Commercial name** | **Final concentration** | **Reference** |
| --- | --- | --- | --- | --- |
|  |  |  |  |  |
| Amino acids | D-Arginine | D-Arginine | 2 mM | A2646 (Sigma) |
|  |  |  |  |  |
|  | L-Arginine | L-Arginine, BioChemika Ultra, ≥ 99.5% |  | A5006 (Sigma-Aldrich) |
|  |  |  |  |  |
|  | L-Tryptophan | L-Tryptophan |  | 108374 (Merck) |
|  |  |  |  |  |
| Plant-derived | Protocatechuate | 3,4-Dihidroxybenzoic acid |  | 37580 (Sigma) |
|  |  |  |  |  |
|  | Pectin | Pectin from citrus peel (Poly-D-galacturonic acid methyl ester) | 1 mg / mL | P9135 (Sigma) |
|  |  |  |  |  |
|  | Humic Acid | Humic acid |  | 53680 (Aldrich) |
|  |  |  |  |  |
