# Supplementary material for: Metabolic versatility of freshwater sedimentary archaea feeding on different organic carbon sources
Source: PLoS One. 2020 Apr 8;15(4):e0231238. doi: 10.1371/journal.pone.0231238 (PMC7141681; doi:10.1371/journal.pone.0231238)
Supplement: S2 Table — In all cases denaturalization stages of 95°C for 20 seconds and annealing steps of 60 seconds were performed. Efficiencies and R2, of the standard curves are displayed as intervals (DOCX) [file pone.0231238.s002.docx]

**Supplementary Table S2.** Primer pairs and conditions used for the quantitative PCR. In all cases denaturalization stages of 95ºC for 20 seconds and annealing steps of 60 seconds were performed. Efficiencies and R^2^, of the standard curves are displayed as intervals.

| **Primer** | **Target group** | **Sequence (5' - 3')** | **Nº cycles** | **Annealing Temperature (ºC)** | **Reference** | **Efficiency (%)** | **R^2^** |
| --- | --- | --- | --- | --- | --- | --- | --- |
| 1048 F | Bacteria | GTGSTGCAYGGYTGTCGTCA | 35 | 60 | [1] | 98.4–99.8 | 0.991–0.999 |
| 1194 R |  | ACGTCRTCCMCACCTTCCTC |  |  |  |  |  |
| 806 F | Archaea | CACAGCGTTTACACCTAG | 40 | 60 | [2] | 97.1–99.3 | 0.993–0.999 |
| 915 R |  | GTGCTCCCCCGCCAATTCCT |  |  | [3] |  |  |
| 242d F | *Bathyarchaeota* | TDACCGGTDCGGGCCGTG | 40 | 68 | [4] | 93.7–97.4 | 0.997–0.999 |
| 678 R |  | AGAACGCGCCCGACGGTG |  |  |  |  |  |
| Thrm-f | *Thermoplasmata* | GGTAAGACGGGTGGC | 40 | 60 | [5] | 89.5–100 | 0.997–0.999 |
| Thrm-r |  | GTATCTAATCCCGTTTGC |  |  |  |  |  |
| *[1] Maeda *et al.*, 2003 [2] Takai *et al.*, 2000 [3] Stahl DA, 1991 [4] Fillol *et al.*, 2015 [5] Compte-Port *et al.*, 2017 | | | | | | | |

**References**

[1] Maeda, H., Fujimoto, C., Haruki, Y., Maeda, T., Kokeguchi, S., Petelin, M. et al. (2003) Quantitative real-time PCR using TaqMan and SYBR Green for *Actinobacillus* *actinomycetemcomitans*, *Porphyromonas gingivalis*, *Prevotella intermedia*, *tetQ* gene and total bacteria. *FEMS Immun. Med. Microbiol.* 39: 81–86.

[2] Takai, K., Horikoshi, K. and Takai, K.E.N. (2000) Rapid detection and quantification of members of the archaeal community by quantitative PCR using fluorogenic probes. *Appl. Environ. Microbiol.* 66: 5066–5072.

[3] Stahl DA, A.R. (1991) Development and application of nucleic acid probes. In, Stackebrandt EGM (ed), Nucleic acid techniques in bacterial systematics. Chincheston UK, pp. 205–248.

[4] Fillol, M., Sànchez-Melsió, A., Gich, F., and Borrego, C.M. (2015) Diversity of Miscellaneous Crenarchaeotic Group archaea in freshwater karstic lakes and their segregation between planktonic and sediment habitats. *FEMS Microbiol. Ecol.* 91: 1–16.

[5] Compte-Port, S., Subirats, J., Fillol, M., Sànchez-Melsió, A., Marcé, R., Rivas-Ruiz, P., et al. (2017) Abundance and co-distribution of widespread marine archaeal lineages in surface sediments of freshwater water bodies across the Iberian Peninsula. *Microb. Ecol.* 74: 776–787.
