# Supplementary material for: Metabolic versatility of freshwater sedimentary archaea feeding on different organic carbon sources
Source: PLoS One. 2020 Apr 8;15(4):e0231238. doi: 10.1371/journal.pone.0231238 (PMC7141681; doi:10.1371/journal.pone.0231238)
Supplement: S3 Table — Sulfide concentration was determined according to Brock et al (J. Bacteriol. 1971, 107:303–314) after fixation of sulfide using zinc acetate (see Material and Methods for details). Values are the mean of replicate measurements ± standard deviation. (DOCX) [file pone.0231238.s003.docx]

**Supplementary Table S3.** Concentration of sulfide in water from the lake bottom, the overlying water carry over with the collected sediment and the rinse water (RW) used for setting-up the experimental microcosms. Sulfide concentration was determined according to Brock et al (*J. Bacteriol.* 1971, 107:303–314) after fixation of sulfide using zinc acetate (see Material and Methods for details). Values are the mean of replicate measurements ± standard deviation.

| Sample | Mean [H_2_S] (µM) |
| --- | --- |
| Bottom water | 725.5 ± 55.37 |
| Overlying water | 400.9 ± 108.8 |
| Rinse Water* | 558.9 ± 194.7 |

* After correction with Na_2_S (see Material and Methods for details)
