# Supplementary material for: Metabolic versatility of freshwater sedimentary archaea feeding on different organic carbon sources
Source: PLoS One. 2020 Apr 8;15(4):e0231238. doi: 10.1371/journal.pone.0231238 (PMC7141681; doi:10.1371/journal.pone.0231238)
Supplement: S4 Table — CNT: Control (no addition of organic carbon); D-Arg: D-Arginine; L-Arg: L-Arginine; Trp: Tryptophan; Ptc: Protocatechuate; HA: Humic Acids; Pec: Pectin. (DOCX) [file pone.0231238.s004.docx]

**Supplementary Table S4.** Mean relative abundance of archaeal groups in libraries from biofilm and sediment after 7 (n=4) and 30 days (n=2) of incubation under treatment conditions. CNT: Control (no addition of organic carbon); D-Arg: D-Arginine; L-Arg: L-Arginine; Trp: Tryptophan; Ptc: Protocatechuate; HA: Humic Acids; Pec: Pectin.

**
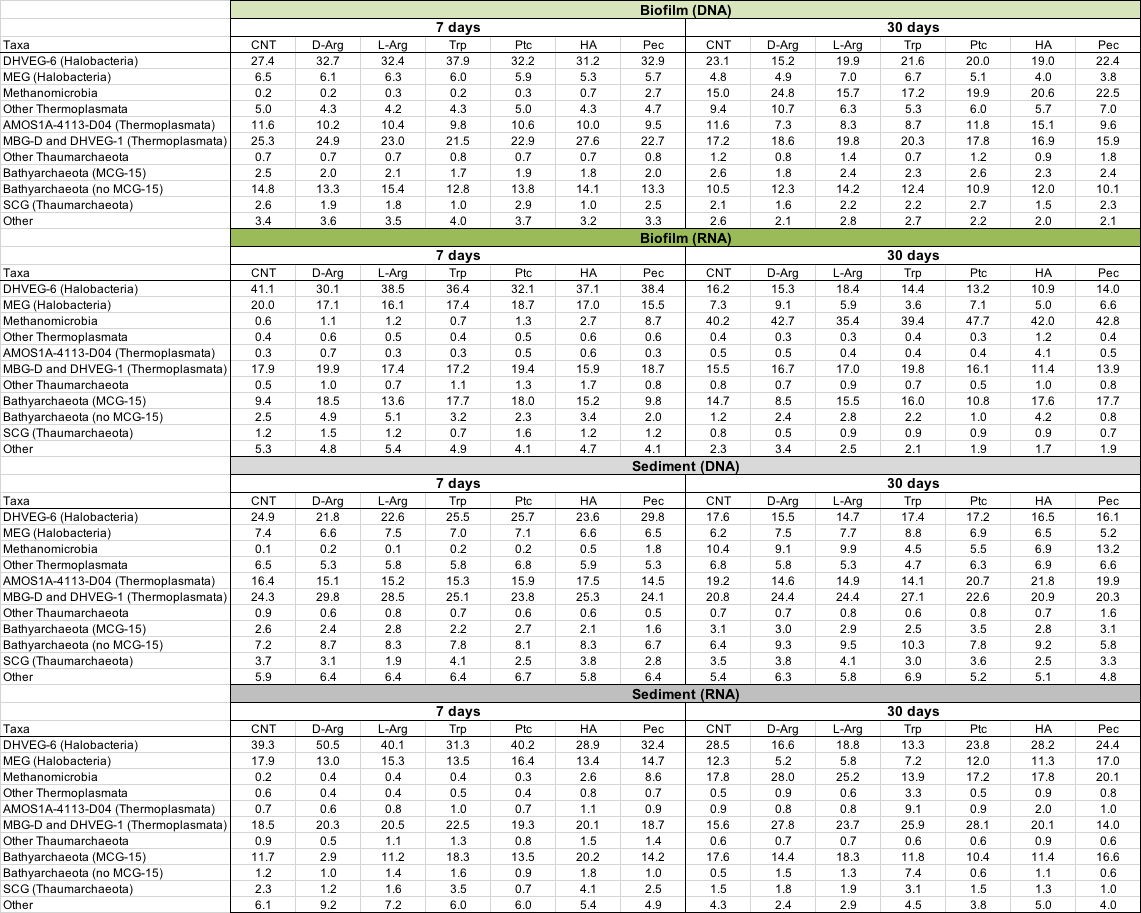
**
