# Supplementary material for: Metabolic versatility of freshwater sedimentary archaea feeding on different organic carbon sources
Source: PLoS One. 2020 Apr 8;15(4):e0231238. doi: 10.1371/journal.pone.0231238 (PMC7141681; doi:10.1371/journal.pone.0231238)
Supplement: S5 Table — Values are displayed as the average ± standard deviation of biological replicates (n = 4 for 7 days and n = 2 for 30 days of incubation) which share same levels across experimental factors. (DOCX) [file pone.0231238.s005.docx]

**Supplementary Table S5.** Copy numbers of the 16S rRNA gene (normalized by dry weight) for Archaea, Bathyarchaeota and Thermoplasmata. Values are displayed as the average ± standard deviation of biological replicates (*n*=4 for 7 days and *n*=2 for 30 days of incubation) which share same levels across experimental factors.

|  |  | **BIOFILM** | | | | **SEDIMENT** | | | |  |
| --- | --- | --- | --- | --- | --- | --- | --- | --- | --- | --- |
|  |  | **DNA fraction** | | **cDNA fraction** | | **DNA fraction** | | **cDNA fraction** | |  |
|  |  | Average | Standard deviation | Average | Standard deviation | Average | Standard deviation | Average | Standard deviation |  |
|  |  |  |  |  |  |  |  |  |  |  |
| 7 days | Control | 2.14 x 10^10^ | 1.12 x 10^10^ | 1.02 x 10^11^ | 7.37 x 10^10^ | 5.49 x 10^9^ | 1.54 x 10^9^ | 2.48 x 10^10^ | 9.65 x 10^9^ | ARCHAEA |
|  | D-Arginine | 1.87 x 10^10^ | 9.72 x 10^9^ | 1.45 x 10^11^ | 2.98 x 10^10^ | 4.92 x 10^9^ | 2.22 x 10^9^ | 6.36 x 10^9^ | 2.01 x 10^9^ |  |
|  | L-Arginine | 1.78 x 10^10^ | 6.56 x 10^9^ | 1.40 x 10^11^ | 2.18 x 10^10^ | 5.00 x 10^9^ | 1.36 x 10^9^ | 1.50 x 10^10^ | 1.07 x 10^10^ |  |
|  | Tryptophan | 1.98 x 10^10^ | 8.78 x 10^9^ | 1.01 x 10^11^ | 3.44 x 10^10^ | 6.13 x 10^9^ | 1.81 x 10^9^ | 1.73 x 10^10^ | 2.88 x 10^9^ |  |
|  | Protocatechuate | 2.06 x 10^10^ | 1.15 x 10^10^ | 1.31 x 10^11^ | 2.49 x 10^10^ | 4.69 x 10^9^ | 1.49E x 10^9^ | 1.93 x 10^10^ | 1.91 x 10^10^ |  |
|  | Humic acids | 2.60 x 10^10^ | 3.03 x 10^9^ | 1.51 x 10^11^ | 4.09 x 10^10^ | 6.28 x 10^9^ | 8.66 x 10^8^ | 2.07 x 10^10^ | 4.79 x 10^9^ |  |
|  | Pectin | 3.27 x 10^10^ | 6.47 x 10^9^ | 1.58 x 10^11^ | 6.72 x 10^10^ | 7.98 x 10^9^ | 4.48 x 10^9^ | 3.66 x 10^10^ | 1.29 x 10^10^ |  |
| 30 days | Control | 1.70 x 10^11^ | 2.31 x 10^10^ | 3.00 x 10^11^ | 3.38 x 10^10^ | 2.12 x 10^10^ | 1.44 x 10^9^ | 3.75 x 10^10^ | 5.16 x 10^9^ |  |
|  | D-Arginine | 2.12 x 10^11^ | 4.25 x 10^10^ | 3.24 x 10^11^ | 3.59 x 10^10^ | 1.11 x 10^10^ | 5.67 x 10^9^ | 3.72 x 10^10^ | 3.68 x 10^8^ |  |
|  | L-Arginine | 1.42 x 10^11^ | 2.74 x 10^9^ | 1.89 x 10^11^ | 2.01 x 10^10^ | 1.92 x 10^10^ | 2.53 x 10^9^ | 3.74 x 10^10^ | 9.74 x 10^9^ |  |
|  | Tryptophan | 1.26 x 10^11^ | 5.73 x 10^10^ | 2.75 x 10^11^ | 7.61 x 10^10^ | 1.26 x 10^10^ | 8.49 x 10^8^ | 2.83 x 10^10^ | 9.66 x 10^9^ |  |
|  | Protocatechuate | 1.52 x 10^11^ | 1.30 x 10^10^ | 3.31 x 10^11^ | 5.16 x 10^10^ | 1.66 x 10^10^ | 4.97 x 10^9^ | 2.66 x 10^10^ | 6.15 x 10^9^ |  |
|  | Humic acids | 9.81 x 10^11^ | 4.31 x 10^9^ | 2.89 x 10^11^ | 5.65 x 10^10^ | 1.88 x 10^10^ | 3.90 x 10^9^ | 1.90 x 10^10^ | 1.74 x 10^10^ |  |
|  | Pectin | 1.28 x 10^11^ | 5.40 x 10^10^ | 2.30 x 10^11^ | 3.41 x 10^10^ | 1.94 x 10^10^ | 6.19 x 10^8^ | 3.39 x 10^10^ | 1.97 x 10^9^ |  |
| 7 days | Control | 7.43 x 10^8^ | 4.33 X 10^8^ | 7.94 X 10^8^ | 6.49 X 10^8^ | 2.19 X 10^8^ | 6.39 X 10^7^ | 5.02 X 10^8^ | 2.01 X 10^8^ | BATHYARCHAEOTA |
|  | D-Arginine | 1.65 X 10^9^ | 9.30 X 10^8^ | 8.97 X 10^8^ | 9.98 X 10^7^ | 2.32 X 10^8^ | 1.49 X 10^8^ | 1.17 X 10^8^ | 8.05 X 10^7^ |  |
|  | L-Arginine | 1.86 X 10^9^ | 1.01 X 10^9^ | 1.42 X 10^9^ | 2.33 X 10^8^ | 2.46 X 10^8^ | 7.58 X 10^7^ | 3.43 X 10^8^ | 2.94 X 10^8^ |  |
|  | Tryptophan | 1.29 X 10^9^ | 7.43 X 10^8^ | 8.03 X 10^8^ | 2.42 X 10^8^ | 2.42 X 10^8^ | 7.38 X 10^7^ | 3.81 X 10^8^ | 1.31 X 10^8^ |  |
|  | Protocatechuate | 7.34 x 10^8^ | 4.79 X 10^8^ | 9.57 X 10^8^ | 4.31 X 10^8^ | 1.79 X 10^8^ | 6.07 X 10^7^ | 4.37 X 10^8^ | 4.61 X 10^8^ |  |
|  | Humic acids | 8.97 X 10^8^ | 1.31 X 10^8^ | 1.34 X 10^9^ | 4.11 X 10^8^ | 2.23 X 10^8^ | 3.38 X 10^7^ | 4.26 X 10^8^ | 1.85 X 10^8^ |  |
|  | Pectin | 9.48 X 10^8^ | 1.52 X 10^8^ | 8.68 X 10^8^ | 2.99 X 10^8^ | 1.81 X 10^8^ | 1.12 X 10^8^ | 3.94 X 10^8^ | 1.99 X 10^8^ |  |
| 30 days | Control | 9.30 X 10^9^ | 8.90 X 10^8^ | 2.97 X 10^9^ | 1.06 X 10^9^ | 2.28 X 10^9^ | 1.17 X 10^8^ | 4.85 X 10^8^ | 7.25 X 10^7^ |  |
|  | D-Arginine | 8.15 X 10^9^ | 5.00 X 10^8^ | 3.63 X 10^9^ | 4.67 X 10^8^ | 1.12 X 10^9^ | 7.28 X 10^8^ | 3.96 X 10^8^ | 6.54 X 10^7^ |  |
|  | L-Arginine | 1.17 x 10^10^ | 2.03 X 10^9^ | 3.26 X 10^9^ | 6.48 X 10^7^ | 1.26 X 10^9^ | 1.99 X 10^8^ | 4.42 X 10^8^ | 6.45 X 10^7^ |  |
|  | Tryptophan | 5.52 X 10^9^ | 3.57 X 10^8^ | 3.06 X 10^9^ | 4.63 X 10^8^ | 7.97 X 10^8^ | 3.02 X 10^7^ | 6.46 X 10^8^ | 2.58 X 10^8^ |  |
|  | Protocatechuate | 6.88 X 10^9^ | 1.22 X 10^9^ | 1.92 X 10^9^ | 1.77 X 10^8^ | 2.22 X 10^9^ | 1.10 X 10^9^ | 3.74 X 10^8^ | 4.04 X 10^7^ |  |
|  | Humic acids | 5.66 X 10^9^ | 1.49 X 10^9^ | 2.71 X 10^9^ | 3.14 X 10^8^ | 1.66 X 10^9^ | 2.40 X 10^8^ | 3.82 X 10^8^ | 3.92 X 10^8^ |  |
|  | Pectin | 6.27 X 10^9^ | 1.78 X 10^9^ | 1.64 X 10^9^ | 1.12 X 10^8^ | 1.08 X 10^9^ | 1.54 X 10^8^ | 3.90 X 10^8^ | 2.74 X 10^6^ |  |
| 7 days | Control | 1.97 X 10^9^ | 1.22 X 10^9^ | 3.88 X 10^8^ | 3.04 X 10^8^ | 4.19 X 10^8^ | 1.09 X 10^8^ | 9.80 X 10^7^ | 4.95 X 10^7^ | THERMOPLASMATA |
|  | D-Arginine | 1.79 X 10^9^ | 9.41 X 10^8^ | 7.73 X 10^8^ | 1.28 X 10^8^ | 4.24 X 10^8^ | 1.96 X 10^8^ | 2.08 X 10^7^ | 6.17 X 10^6^ |  |
|  | L-Arginine | 1.61 X 10^9^ | 7.36 X 10^8^ | 5.98 X 10^8^ | 1.18 X 10^8^ | 4.57 X 10^8^ | 1.26 X 10^8^ | 7.91 X 10^7^ | 8.34 X 10^7^ |  |
|  | Tryptophan | 2.06 X 10^9^ | 1.36 X 10^9^ | 4.41 X 10^8^ | 1.65 X 10^8^ | 4.65 X 10^8^ | 9.66 X 10^7^ | 1.11 X 10^8^ | 9.08 X 10^7^ |  |
|  | Protocatechuate | 2.03 X 10^9^ | 1.41 X 10^9^ | 4.97 X 10^8^ | 1.32 X 10^8^ | 3.38 X 10^8^ | 1.19 X 10^8^ | 9.33 X 10^7^ | 1.00 X 10^8^ |  |
|  | Humic acids | 2.42 X 10^9^ | 2.27 X 10^8^ | 7.15 X 10^8^ | 2.84 X 10^8^ | 4.53 X 10^8^ | 6.29 X 10^7^ | 8.70 X 10^7^ | 8.34 X 10^6^ |  |
|  | Pectin | 3.04 X 10^9^ | 5.58 X 10^8^ | 4.39 X 10^8^ | 2.19 X 10^8^ | 3.92 X 10^8^ | 2.47 X 10^8^ | 1.09 X 10^8^ | 4.15 X 10^7^ |  |
| 30 days | Control | 2.18 X 10^9^ | 2.40 X 10^8^ | 1.11 X 10^9^ | 4.00 X 10^8^ | 3.57 X 10^8^ | 7.01 X 10^7^ | 1.60 X 10^8^ | 1.66 X 10^7^ |  |
|  | D-Arginine | 1.70 X 10^9^ | 2.40 X 10^7^ | 1.11 X 10^9^ | 1.19 X 10^8^ | 2.11 X 10^8^ | 9.12 X 10^7^ | 1.42 X 10^8^ | 2.59 X 10^7^ |  |
|  | L-Arginine | 1.87 X 10^9^ | 3.24 X 10^8^ | 7.75 X 10^8^ | 1.02 X 10^8^ | 3.58 X 10^8^ | 2.65 X 10^7^ | 1.52 X 10^8^ | 3.71 X 10^7^ |  |
|  | Tryptophan | 1.12 X 10^9^ | 9.97 X 10^7^ | 9.41 X 10^8^ | 1.27 X 10^8^ | 2.29 X 10^8^ | 4.47 X 10^6^ | 2.04 X 10^8^ | 9.70 X 10^7^ |  |
|  | Protocatechuate | 1.65 X 10^9^ | 1.74 X 10^8^ | 6.44 X 10^8^ | 4.00 X 10^7^ | 3.72 X 10^8^ | 1.26 X 10^8^ | 1.07 X 10^8^ | 1.60 X 10^7^ |  |
|  | Humic acids | 1.19 X 10^9^ | 1.05 X 10^8^ | 7.77 X 10^8^ | 1.17 X 10^8^ | 3.45 X 10^8^ | 4.09 X 10^7^ | 9.32 X 10^7^ | 9.81 X 10^7^ |  |
|  | Pectin | 1.40 X 10^9^ | 4.99 X 10^8^ | 8.96 X 10^8^ | 1.83 X 10^8^ | 3.02 X 10^8^ | 4.65 X 10^7^ | 2.18 X 10^8^ | 2.12 X 10^7^ |  |
