# Supplementary material for: Metabolic versatility of freshwater sedimentary archaea feeding on different organic carbon sources
Source: PLoS One. 2020 Apr 8;15(4):e0231238. doi: 10.1371/journal.pone.0231238 (PMC7141681; doi:10.1371/journal.pone.0231238)
Supplement: S1 Fig — (DOCX) [file pone.0231238.s006.docx]

**Suppl. Figure S1:** Depth profile of temperature (red circles), conductivity (black squares), dissolved oxygen (blue circles), oxidation-reduction potential (grey triangles) and sulfide concentration (yellow diamonds) in Lake Cisó at the day of sampling (29/01/2015).
