# Supplementary material for: Metabolic versatility of freshwater sedimentary archaea feeding on different organic carbon sources
Source: PLoS One. 2020 Apr 8;15(4):e0231238. doi: 10.1371/journal.pone.0231238 (PMC7141681; doi:10.1371/journal.pone.0231238)
Supplement: S2 Fig — RW: rinse water (see main text for details). (DOCX) [file pone.0231238.s007.docx]

**Suppl. Figure S2:** Schematic representation of the sampling procedures, experimental setup for the preparation of microcosms and the list of carbon compounds (treatments). RW: rinse water (see main text for details).
