# Supplementary material for: Metabolic versatility of freshwater sedimentary archaea feeding on different organic carbon sources
Source: PLoS One. 2020 Apr 8;15(4):e0231238. doi: 10.1371/journal.pone.0231238 (PMC7141681; doi:10.1371/journal.pone.0231238)
Supplement: S3 Fig — PCoA Ordination of samples according to (A) unweighted and (B) weighted UniFrac distance using forward (5’) and reverse (3’) sequences. Samples are coloured according to substratum, biofilm (red) and sediment (blue). Samples derived from forward and reverse sequencing are linked with a bar: in every case, the distance between the 5′ and 3′ reads of the same samples is much smaller than the distance between samples. Results from the Procrustes analysis are also shown for each case (10,000 Monte Carlo simulations). (DOCX) [file pone.0231238.s008.docx]

**Suppl. Figure S3:** PCoA Ordination of samples according to (A) unweighted and (B) weighted UniFrac distance using forward (5’) and reverse (3’) sequences. Samples are coloured according to substratum, biofilm (red) and sediment (blue). Samples derived from forward and reverse sequencing are linked with a bar: in every case, the distance between the 5′ and 3′ reads of the same samples is much smaller than the distance between samples. Results from the Procrustes analysis are also shown for each case (10,000 Monte Carlo simulations).
