# Supplementary material for: Metabolic versatility of freshwater sedimentary archaea feeding on different organic carbon sources
Source: PLoS One. 2020 Apr 8;15(4):e0231238. doi: 10.1371/journal.pone.0231238 (PMC7141681; doi:10.1371/journal.pone.0231238)
Supplement: S4 Fig — Composition archaeal communities in (A) biofilm and (B) sediment samples used as inoculum of experimental microcosms. The relative abundance of each taxon is depicted as a percentage of total reads. (DOCX) [file pone.0231238.s009.docx]

**
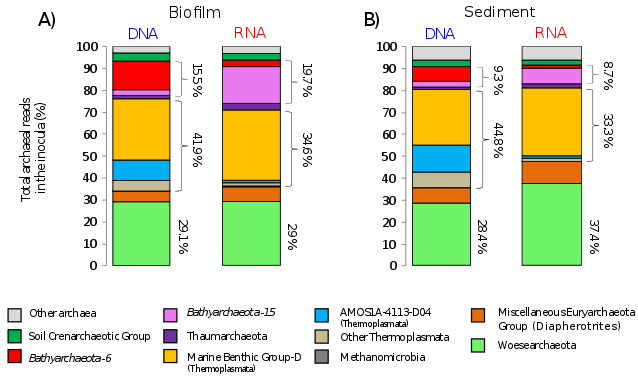
**

**Suppl. Figure S4:** Composition archaeal communities in (A) biofilm and (B) sediment samples used as inoculum of experimental microcosms. The relative abundance of each taxon is depicted as a percentage of total reads.
