# Supplementary material for: Metabolic versatility of freshwater sedimentary archaea feeding on different organic carbon sources
Source: PLoS One. 2020 Apr 8;15(4):e0231238. doi: 10.1371/journal.pone.0231238 (PMC7141681; doi:10.1371/journal.pone.0231238)
Supplement: S5 Fig — Comparison of alpha diversity estimators (number of OTUs and Shannon index) of biofilm and sediment archaeal communities between (a) DNA and RNA libraries; (b) between niches (biofilm vs. sediment) for archaeal communities in the RNA library; (c) incubation time (0, 7, and 30 days) for biofilm communities in the RNA library; (d) incubation time (0, 7, and 30 days) for sediment communities in the RNA library. Significant differences for all comparisons have been assessed by Mann-Whitney test and indicated by letters above boxplots with correspondent p values. (DOCX) [file pone.0231238.s010.docx]

**Suppl. Figure S5.** Comparison of alpha diversity estimators (number of OTUs and Shannon index) of biofilm and sediment archaeal communities between (a) DNA and RNA libraries; (b) between niches (biofilm *vs.* sediment) for archaeal communities in the RNA library; (c) incubation time (0, 7, and 30 days) for biofilm communities in the RNA library; (d) incubation time (0, 7, and 30 days) for sediment communities in the RNA library. Significant differences for all comparisons have been assessed by Mann-Whitney test and indicated by letters above boxplots with correspondent *p* values.
