# Supplementary material for: Metabolic versatility of freshwater sedimentary archaea feeding on different organic carbon sources
Source: PLoS One. 2020 Apr 8;15(4):e0231238. doi: 10.1371/journal.pone.0231238 (PMC7141681; doi:10.1371/journal.pone.0231238)
Supplement: S6 Fig — Comparison has been done according to: (a) nucleic acids; (b) habitat (only the archaeal community in the RNA library); (c); incubation time (only the biofilm archaeal community in the RNA library); (d) incubation time (only the sediment archaeal community in the RNA library). Significant differences for all comparisons have been assessed by PERMANOVA and beta dispersion analyses (9,999 permutations). p-values of both tests are shown at the bottom right of each plot, together with the amount of variance (%) explained by each grouping. Prominent archaeal lineages contributing to such differences have been identified using SIMPER analysis (9,999 permutations) and results are shown in the top right of each plot when appropriate. (DOCX) [file pone.0231238.s011.docx]

**Suppl. Figure S6.** Non-metric multidimensional scaling (NMDS) ordination of samples according to community composition (Bray-Curtis dissimilarity distance). Comparison has been done according to: (a) nucleic acids; (b) habitat (only the archaeal community in the RNA library); (c); incubation time (only the biofilm archaeal community in the RNA library); (d) incubation time (only the sediment archaeal community in the RNA library). Significant differences for all comparisons have been assessed by PERMANOVA and beta dispersion analyses (9,999 permutations). *p-*values of both tests are shown at the bottom right of each plot, together with the amount of variance (%) explained by each grouping. Prominent archaeal lineages contributing to such differences have been identified using SIMPER analysis (9,999 permutations) and results are shown in the top right of each plot when appropriate.
