# Supplementary material for: Metabolic versatility of freshwater sedimentary archaea feeding on different organic carbon sources
Source: PLoS One. 2020 Apr 8;15(4):e0231238. doi: 10.1371/journal.pone.0231238 (PMC7141681; doi:10.1371/journal.pone.0231238)
Supplement: S8 Fig — Samples are colored according to the carbon source, namely: amino acids (grey squares), plant-derived compounds (black squares) and no carbon addition (white squares). p-values from the PERMANOVA and the beta dispersion tests and the amount of explained variance by amendment type (%) are also shown. See main text for details. (DOCX) [file pone.0231238.s013.docx]

**Suppl. Figure S8.** Non-metric multidimensional scaling (NMDS) ordination of samples from microcosms inoculated with sediment material according to the abundance of OTUs affiliated to MBG-D (upper panels) and Woesearchaeota (lower panels) in RNA libraries after 7 days (left) or 30 days (right) of incubation. Samples are colored according to the carbon source, namely: amino acids (grey squares), plant-derived compounds (black squares) and no carbon addition (white squares). *p*-values from the PERMANOVA and the beta dispersion tests and the amount of explained variance by amendment type (%) are also shown. See main text for details.
